# Bilingualism and working memory performance: Evidence from a large-scale online study

**DOI:** 10.1371/journal.pone.0205916

**Published:** 2018-11-02

**Authors:** Karolina M. Lukasik, Minna Lehtonen, Anna Soveri, Otto Waris, Jussi Jylkkä, Matti Laine

**Affiliations:** 1 Department of Psychology, Åbo Akademi University, Turku, Finland; 2 Cognitive Brain Research Unit, Department of Psychology and Logopedics, Faculty of Medicine, University of Helsinki, Helsinki, Finland; 3 MultiLing Center for Multilingualism in Society across the Lifespan, Department of Linguistics and Scandinavian studies, University of Oslo, Oslo, Norway; 4 Turku Brain and Mind Center, University of Turku, Turku, Finland; University of St Andrews, UNITED KINGDOM

## Abstract

The bilingual executive advantage (BEA) hypothesis has attracted considerable research interest, but the findings are inconclusive. We addressed this issue in the domain of working memory (WM), as more complex WM tasks have been underrepresented in the previous literature. First, we compared early and late bilingual vs. monolingual WM performance. Second, we examined whether certain aspects of bilingual experience, such as language switching frequency, are related to bilinguals’ WM scores. Our online sample included 485 participants. They filled in an extensive questionnaire including background factors such as bilingualism and second language (L2) use, and performed 10 isomorphic verbal and visuospatial WM tasks that yielded three WM composite scores (visuospatial WM, verbal WM, n-back). For verbal and visuospatial WM composites, the group comparisons did not support the BEA hypothesis. N-back analysis showed an advantage of late bilinguals over monolinguals and early bilinguals, while the latter two groups did not differ. This between-groups analysis was followed by a regression analysis relating features of bilingual experience to n-back performance, but the results were non-significant in both bilingual groups. In sum, group differences supporting the BEA hypothesis were limited only to the n-back composite, and this composite was not predicted by bilingualism-related features. Moreover, Bayesian analyses did not give consistent support for the BEA hypothesis. Possible reasons for the failure to find support for the BEA hypothesis are discussed.

## Introduction

Bilinguals’ possible advantage over monolinguals in executive tasks (bilingual executive advantage, BEA) has been a topic of intensive research interest and growing controversy especially during the past decade [1–6]. Earlier studies appeared to paint a relatively consistent picture where bilinguals performed better than monolinguals on a range of mostly nonverbal tasks calling for executive functions, such as conflict resolution [7,8] and task switching [9]. BEA has been assumed to stem from the lifelong experience of managing two languages that requires inhibition of the currently irrelevant language, switching between languages, and monitoring the language context to choose the appropriate target language [10]. As bilingual experience varies, e.g., depending on age of acquisition of the second language, language proficiency and time spent using both languages, so may also its effects on executive function [11].

However, the earlier studies supporting the BEA hypothesis have been challenged by several more recent reports that have failed to replicate these findings [12–15], and the existence of BEA has been questioned [3,4]. The field has also evidenced a publication bias, with null or negative findings being published less often than positive ones [6]. After controlling for publication bias, a recent extensive meta-analysis on 152 studies on adults found no systematic support for BEA in any of the six studied executive domains (inhibitory control, set shifting, monitoring, working memory, attention, verbal fluency) [16].

One important cognitive domain where the putative BEA has received increasing research interest during the last decade is working memory (WM). It is a temporary memory system that acts as a constantly updated “mental platform” for information relevant for current activities and goals that we are focusing at. WM skills have been linked to many important aspects of everyday life, ranging from language learning and academic achievement to mental health (for example, see [17,18]). In the area of bilingualism, a possible enhancement of WM could stem from the constant “juggling” of two languages in the mind [19] which calls for WM resources and their efficient allocation [20]. From this perspective, bilinguals’ lifelong practice of managing two languages could enhance WM capacity or efficiency.

As with other cognitive domains, results from individual studies contrasting bilinguals’ and monolinguals’ WM performance have so far been inconsistent. In recent years, four meta-analyses have addressed the putative bilingual advantage in WM [21–23, 16]. Adesope and colleagues [21], investigating the effects of bilingualism on cognition in children and young adults, pooled together various WM measures from four studies and found a moderate BEA effect (g = .48). Grundy and Timmer [22] reported a small to medium population effect (ρ = .2) in their meta-analysis of 27 studies conducted on both children and adults, leading the authors to conclude that bilinguals have a small advantage over monolinguals in WM. Grundy and Timmer also performed moderator analyses on age group (children, young adults, older adults), task type (verbal vs. nonverbal), and task language for the verbal WM tasks (dominant L1 vs. second language, L2). These analyses indicated the greatest WM advantage for children as compared to young and older adults. The analysis showed no effect of task type on the magnitude of the BEA, but a greater advantage when the bilinguals were completing a verbal WM task in their L1 than in L2. Grundy and Timmer [22] attribute the larger BEA in children’s WM performance to the high neurocognitive demands that children face when learning a new language. However, they did not study whether the outcomes depended on matching of the bilingual and monolingual samples on socioeconomic status (SES) or education. This issue is of particular importance, as SES and education have been shown to influence WM performance [24,25], but unfortunately, studies comparing monolingual and bilingual groups do not always mention whether the groups were matched according to these pertinent background factors. The meta-analysis by von Bastian and colleagues found a small but positive effect (g = .11) of bilingualism on WM performance. However, they also report great heterogeneity in the data, but no task- or background-related moderators that could account for the variability [23].

The fourth and most recent meta-analysis by Lehtonen and colleagues [16] analyzed 251 effect sizes from four separate WM paradigms (complex span, simple span, letter-number sequencing, and n-back) and found a very small positive effect of bilingualism on WM performance (Hedge’s g = .07), which disappeared after correcting for the observed publication bias. It also became nonsignificant when including only original studies that had explicitly matched the participant groups, e.g., with regard to education. The comparison between verbal and nonverbal WM tasks revealed that the effect size was smaller for verbal tasks (Hedge’s g = .01) than for nonverbal tasks (Hedge’s g = .30). However, when correcting for bias in this analysis, even the nonverbal advantage vanished. In the moderator analysis on the role of L2 age of acquisition (AoA), it was found that early acquirers (i.e., AoA = < 6 years) showed a small WM-related bilingual advantage (Hedge’s g = .23, p < .01) that late acquirers did not exhibit (Hedge’s g = .02, p = .735), but also this advantage disappeared when correcting for publication bias. No moderating effects on WM were found for L2 proficiency.

All in all, three of the four earlier meta-analyses [21–23] suggested that there is a small-to-moderate positive effect of bilingualism on WM. Two of those studies [21, 22] also found evidence of a publication bias, while von Bastian and colleagues [23] report no asymmetry between the publication of positive and negative results. In turn, the most recent and largest meta-analysis to date by Lehtonen and colleagues [16] found no positive effect in adults after correcting for the asymmetry between positive and negative results. However, one should note that the previous studies on WM and thus the data available for meta-analyses have been dominated by simple span tasks, and more taxing and thereby potentially more sensitive WM tasks (n/back, complex span, letter-number sequencing) have been utilized in fewer studies. Moreover, WM studies using verbal tasks may have been affected by bilinguals’ general disadvantage on verbal measures when compared with monolingual speakers [26,27]. Thus, further research with a wider range of WM tasks is warranted.

It is important to highlight that bilingualism represents a spectrum rather than a categorical variable [11,28,29]. Balanced bilingualism, or equilingualism, is a rare phenomenon, and language usage can change throughout a person’s lifetime in a dynamic fashion. Therefore, in this study, we defined bilingualism in a broad sense as the ability to speak two languages–regardless of AoA, proficiency, and recent language use–while acknowledging that these factors, among others, characterize individual bilingual experience. This large individual variability in bilinguals also opens the way to explore the associations between features of bilingual experience and level of executive function. The latter correlative approach can be seen as a complement to bilingual-monolingual contrasts that directly address BEA. If some features of bilingual experience indeed enhance executive function, it should be possible to find positive correlations between those features and executive task performance. For example, one could hypothesize that earlier AoA would predict better executive task performance, as a person would have had longer practice in using that language and, by extension, more executive training [30]. Another general feature of bilingual experience, L2 proficiency, might also modulate level of executive task performance: it has been suggested that higher L2 proficiency could pose more demands on interference management and lead to better EF [31, 32], or that lower L2 proficiency would necessitate stronger inhibition of L1 [33–35]. With regard to more qualitative features of bilingual experience that might enhance executive functions, one prominent candidate is the frequency of switching between two languages [36], as it arguably requires suppressing one language and activating the other [9, 37]. Thus, bilinguals who switch more often might show better executive task performance on, e.g., complex WM tasks. This idea, however, has been challenged by studies that found no link between frequency of language switching and the commonly used measures of resolving incongruency on executive tasks [15, 38], and it has been suggested that language switching does not interact with general executive mechanisms in a consistent fashion [39].

Thus far, possible associations between features of bilingual experience and WM performance have been explored only in a limited number of studies. In 6-year-old bilingual children, Blom and colleagues [40] found that higher bilingual proficiency was related to a better performance on a backward digit recall task. Tse and Altarriba [41] found that higher L2 proficiency in bilingual children predicted better verbal WM. While the child research cited above provides some support to the BEA hypothesis, a study by Soveri and colleagues [36] with bilingual adults failed to find significant correlations between the n-back effect and frequency of language switching (as measured by the Bilingual Switching Questionnaire developed by Rodríguez-Fornells and colleagues [42]), L2 AoA, or frequency of everyday use of both languages. Neither did Jylkkä and colleagues [39] find support to the BEA hypothesis in the domain of WM: in their study of adult bilinguals, higher rates of a subtype of language switches, unintended switches, were associated with worse WM updating as measured by n-back. Unintended switches may simply reflect lapses in cognitive control. All in all, there is conflicting evidence concerning the relationships between bilingual experience and executive functions including WM. However, the limited number of existing studies prompts further experiments, especially because such relationships, if they are found, could provide hints on the underlying cognitive mechanisms of the putative BEA.

### The present study

In the present study, we set out to investigate the associations between bilingualism and WM in large, diverse, and well-matched groups of monolinguals, early bilinguals, and late bilinguals. We measured WM with three composite scores that reflect the previously reported latent structure of WM in this sample [43]. To the best of our knowledge, this is the first study that combines the use of an extensive WM task battery and composite scores based on the latent structure of the WM tasks in a study of BEA. This is relevant because individual WM tasks tend to have low intercorrelations and cannot be used interchangeably [44, 45]. We employed two types of analyses. First, we tested directly the BEA hypothesis by comparing WM performance in monolinguals, early bilinguals, and late bilinguals. The BEA hypothesis predicts that bilinguals would outperform monolinguals on WM measures. The difference might be larger for early bilinguals whose longer bilingual experience would have provided more executive practice in managing, switching and monitoring two languages; on the other hand, some studies suggest that for late and unbalanced bilinguals, controlling both languages is more taxing on the EF[33, 37]. Second, we explored whether certain bilingualism-related factors (L2 AoA, L2 proficiency, amount of language switching) would predict WM performance within the bilinguals. If BEA stems from bilingual experience, some crucial aspects of that experience should correlate with measures of executive performance such as success on WM tasks.

## Methods

### Ethics statement

The study was approved by the Joint Ethics Committee at the Departments of Psychology and Logopedics, Åbo Akademi University. Informed consent was obtained from all participants, participation was anonymous, and all participants were informed of their right to withdraw from the study at any time. They received a $10 payment for their participation.

### Participants and procedure

We recruited the participants through the Amazon Mechanical Turk crowdsourcing site [46]and the data (questionnaires and WM measures) were collected using an in-house Java-based test platform. This study used the same data as Waris and colleagues [43]). Overall, 711 U.S. American adult participants completed the study. We excluded 43 participants due to having reported using external tools during task performance, missing data, or taking over 24 hours to complete the study. As depressive symptoms may affect WM performance (see for[47]), we excluded the 136 participants whose scores on the Quick Inventory of Depressive Symptomatology (QIDS;[48]) corresponded to moderate, severe or very severe symptom occurrence, as well as 16 participants who had missing data on the QIDS. Furthermore, we excluded 13 participants whose scores were outliers in the WM tasks according to Mahalanobis distance [49]. At this point, the sample consisted of 503 participants.

We further excluded participants who declared that English was their L2, as we wanted the participants to complete tasks in their first language [16, 22]. We also excluded participants who gave “Latin” (or nonsensical answers) as their L2; however, language proficiency in either L1 or L2 was not an exclusion criterion. These criteria resulted in excluding 7 people. We then excluded 11 participants with missing data on either L2 AoA or L2 proficiency. The final sample comprised 485 participants.

Out of the 485 participants, 265 spoke a second language. 115 reported having learned L2 in the first 12 years of their life, which identified them as early bilinguals. The 150 participants who had acquired their L2 after the age of 12 were classified as late bilinguals. Demographic characteristics of the three language groups (monolinguals, early bilinguals and late bilinguals) are given in Table 1.

**Table 1:** Summary of the background characteristics of the sample.

	Monolinguals	Early bilinguals (AoA ≤ 12)	Late bilinguals (AoA > 12)
n	220	115	150
Mean age (SD)	35.2 (11)	31.9 (10)	33.6 (10)
Gender	56.8% women	56.5% women	57.3% women
Education			
1 (primary education)	1.4%	0%	.7%
2 (lower secondary education)	1.4%	.9%	.7%
3 (higher secondary education)	26.4%	17.4%	18%
4 (basic vocational education)	6.8%	2.6%	9.3%
5 (vocational university/other upper vocational education)	15.9%	8.7%	14%
6 (university: bachelor’s or master’s degree)	46.4%	68.7%	52.7%
7 (university: doctoral degree)	1.8%	1.7%	4.7%
Race & ethnicity			
Hispanic	5.5%	16.5%	3.3%
Black	8.2%	7.8%	8%
White	90.5%	68.7%	88%
Asian	3.2%	23.5%	2.7%
American Indian	1.8%	1.7%	2%
Other / biracial	.9%	4.3%	2%
Employment			
Employed	66.8%	65.2%	74.7%
Student	12.7%	28.7%	20.7%

For WM assessment, we used ten tasks: simple span tasks (backward and forward), complex span tasks, running memory tasks, and 2-back tasks. Each task had two isomorphic versions (numerical-verbal and visuospatial; for details, see [43]). Based on the factor analyses by Waris and colleagues [43], we calculated three composite accuracy scores reflecting the following latent factors: verbal WM (4 tasks), visuospatial WM (4 tasks), and n-back (2 tasks). The scores were Z-transformed and then summed and averaged.

To investigate the language background of the participants, we used several questions (see Table 2). First, the participants were asked to list all the languages they had learned or studied (including the native language) as well as AoA and self-reported proficiency (on a Likert-type scale ranging from 1—*Beginner*, to 6—*Native-level mastery*) for each language. Finally, one question assessed bilingual language switching frequency, tapping the overall number of switches (*On average*, *I switch between different languages [x] times a day*. The response was given on a Likert-type 1–5 scale where 1 = 0–2 switches, 2 = 3–10 switches, 3 = 11–30 switches, 4 = 31–60 switches, and 5 = over 60 daily switches.

**Table 2:** Bilingual language experience variables in the two bilingual groups.

	Early bilinguals (AoA ≤ 12)	Late bilinguals (AoA >12)
n	115	150
Mean L2 AoA (SD)	6 (4.4)	17.7 (6.2)
Mean L2 proficiency (SD)	3.48 (1.8)	2.2 (1.1)
Languge switching frequency (SD)	1.47 (.85)	1.1 (.51)

L2 AoA, self-reported proficiency, percentage of L2 use in the last two years, and switching are reported in Table 2. The most common second languages were Spanish (141 speakers), French (41 speakers) and German (20 speakers).

## Results

### Comparison of background factors between language groups

We investigated whether there were significant differences in the background factors age, education, childhood socioeconomic status and gender between the three groups. A main effect of age was found [F(2,484) = 3.85, p < .05]: mean age in the monolingual group was significantly higher than in the early bilingual group (p < .01), but there was no significant difference between early and late bilingual groups, or between late bilinguals and monolinguals. We also noted a main effect of education [F(2,484) = 6.16, p < .01]. Post-hoc tests revealed that the monolingual group differed significantly from both the early and late bilingual groups, being on average less educated (p < .05). There were no significant differences between the two bilingual groups. The three groups did not differ significantly as regards childhood socioeconomic status [F(2,482) = .994, p = .37]. There was no significant difference between the groups as regards gender [χ^2^(4) = 3.23, p = .52].

### Sample matching

Due to the significant differences between groups in background variables, we decided to use a matching procedure to ensure that the covariates were distributed equally between the three language groups. We used the MatchIt package for statistical software R [50, 51]. MatchIt enables researchers to match two samples within one study according to select variables, and allows a variety of matching algorithms to be used. Whereas matching based on Mahalanobis distance or Propensity Score Matching would usually be the method of choice, we could not apply it since the assumption of normal distribution was not met in our data, and thus the use of those standard procedures might increase bias in the data rather than limit it [52]. Therefore, we applied a so-called genetic matching procedure that uses an evolutionary algorithm to create a set of solutions and determine which one produces the best match[52, 53]. This procedure matches groups on variables of choice by dropping out participants in the groups as well as by ascribing weights to each participant. The weights are used to correct for unequal variances and counteract heteroscedasticity in the data. This method has been successfully used in social sciences, most notably in the re-analysis of the National Supported Work Demonstration Program job training experiment [54, 55].

For the genetic matching procedure, we treated both early and late bilinguals as the reference group, and monolinguals as the matched group. We matched the groups on age and education simultaneously. We did not consider gender or childhood SES as basis for matching, since there was no difference on those variables between the unmatched groups. MatchIt allows the user to specify the parameters of matching, and we chose to discard data from both reference and matched groups if it ensured the best fit. The matching resulted in leaving out 44 participants from the monolingual group and ascribing weights (later used in analyses as weighted least squares) to each participant. Thus, the final matched sample consisted of 176 monolinguals, 115 early bilinguals and 150 late bilinguals. The three groups did not differ significantly on any of the background variables (p’s > .1).

### Descriptive data

The means and standard deviations on the three WM composite measures for the monolingual, early bilingual and late bilingual groups are reported in Table 3. To see if education and childhood SES correlated with WM performance in our sample [24, 25], we investigated Pearson’s correlations between age, education, childhood SES and the three WM composite scores (see Table 4). Age correlated negatively with the visuospatial WM and n-back composites. Education or childhood SES did not correlate significantly with any of the WM measures.

**Table 3:** Mean WM performance on the three composite scores in the monolingual, early bilingual and late bilingual groups. All scores have been z-transformed, then summed and averaged. Higher positive values indicate better performance.

	Monolinguals (n = 176)	Early bilinguals (n = 115)	Late bilinguals (n = 150)
Mean verbal WM score (SD)	-.04 (.77)	.11 (.73)	.11 (.72)
Mean visuospatial WM score (SD)	-.07 (.76)	.13 (.77)	.17 (.74)
Mean n-back score (SD)	-.06 (.87)	-.01 (.85)	.24 (.87)

**Table 4:** Pearson’s correlations between age, education, childhood SES and WM scores in the matched sample (n = 441).

	Age	Education	Childhood SES	Verbal WM	Visuospatial WM
Age	-				
Education	.18**	-			
BF₁₀	90.4	-			
Childhood SES	.03	.12*	-		
BF₁₀	.075	1.43	-		
Verbal WM	-.02	.09	.005	-	
BF₁₀	.07	.37	.08	-	
Visuospatial WM	-.13**	.04	.01	.55**	-
BF₁₀	2.05	.09	.06	>100	-
N-back	-.15**	0	0	.55**	.41**
BF₁₀	10.35	.06	.06	>100	>100

*p < .05;

**p < .01.

p-values are Bonferroni-corrected.

### Differences in language proficiency and language use between early and late bilinguals

We tested whether the two bilingual groups were equal as regards language proficiency, frequency of L2 use and language switching. T-tests between early and late bilinguals revealed significant differences between these two groups. Early bilinguals were significantly more proficient in their L2 (t(263) = 6.94, p < .001). They also used their L2 significantly more often (t(262) = 4.36, p < .001), and declared more frequent switching between languages (t(140) = 4.19, p < .001).

### Visuospatial WM performance in the three language groups

**Fig 1:**
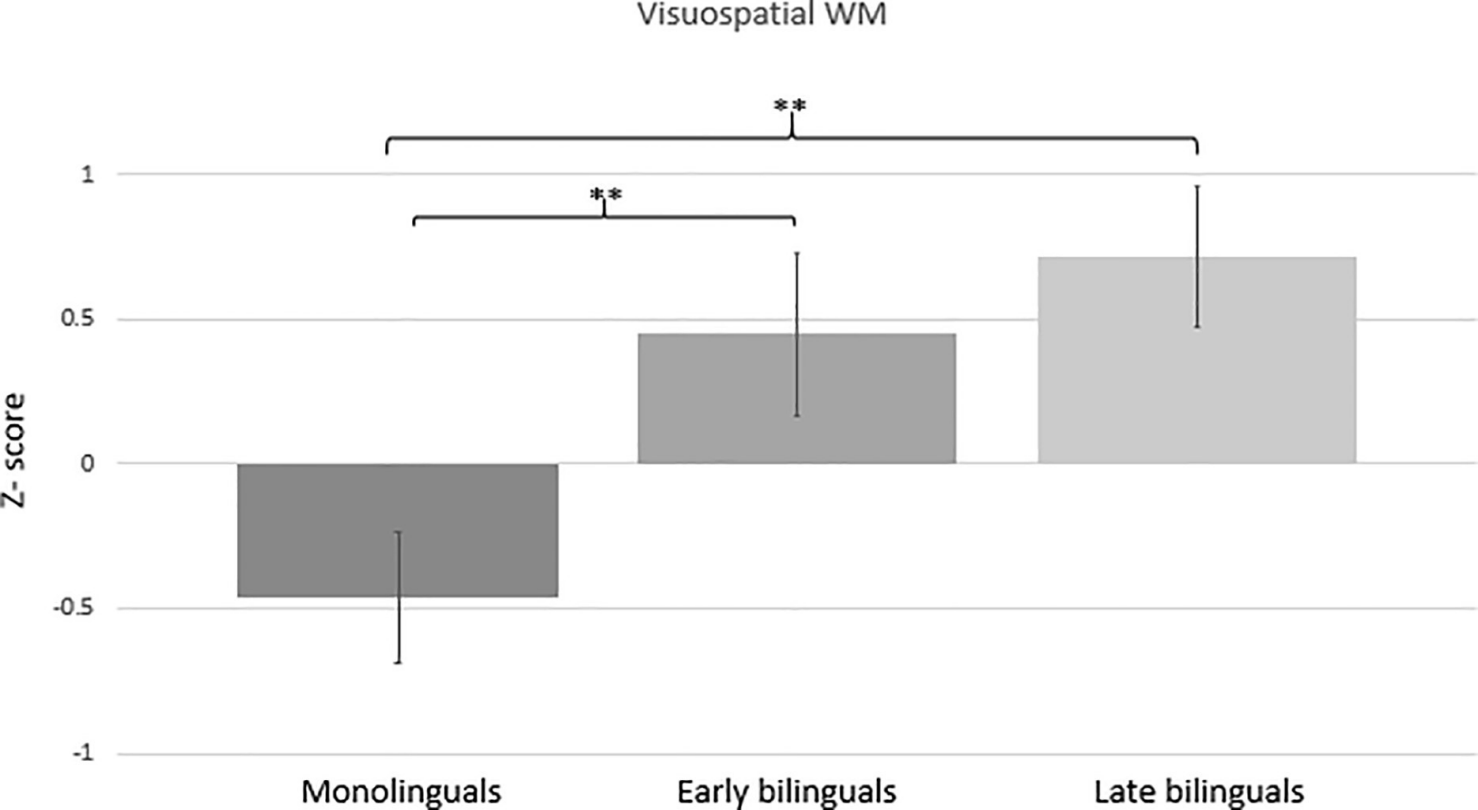
Mean visuospatial WM performance in the three language groups. Bars represent standard errors.

To explore possible differences on visuospatial WM between the three language groups, we conducted an ANCOVA with the visuospatial WM composite as the dependent variable. Age, education and childhood SES served as covariates, and language group (monolingual; early bilingual; late bilingual) as the independent variable. Weights ascribed by the MatchIt algorithm were used as weighted least squares. The analysis revealed a significant main effect of language group [F(2,435) = 6.91, p < .01; partial η^2^ = .031], stemming from higher accuracy scores in the two bilingual groups compared to the monolinguals. Age was a significant covariate (p < .01), with higher age being related to a lower visuospatial WM performance. Pairwise comparisons revealed a significant mean difference between monolinguals and early bilinguals (p < .01) and between monolinguals and late bilinguals (p < .01). The difference between early and late bilinguals was not statistically significant (p > .05). The group differences are shown in Fig 1. In order to obtain a more informative measure of how strongly the data supported the hypothesis of a difference between the language groups, we conducted a Bayesian ANCOVA. The Bayes Factor for language group compared to a null model with just the covariates was BF_10_ = 2.46, indicating that the data provided only anecdotal evidence for the hypothesis that there is a difference in visuospatial WM between the language groups [56].

### Verbal WM performance in the three language groups

We conducted an ANCOVA with the verbal WM composite as the dependent variable, age, education and childhood SES as covariates, and language group (monolingual; early bilingual; late bilingual) as the independent variable. As previously, MatchIt weights were used as weighted least squares. Childhood SES was a significant covariate (p < .05): lower childhood SES was associated with better WM performance. However, a closer examination revealed that this effect was driven by the disproportionate distribution of responses across SES categories. The covariate education was near-significant (p = .057); higher education tended to be associated with better verbal WM performance. The effect of language group was not significant, (F(2,435) = 1.89, p = .15). Bayesian ANCOVA yielded a Bayes Factor of BF_10_ = .04 for the effect of language group, that is, there was substantial evidence for the null hypothesis that there is no difference between the language groups (BF_01_ = 25).

### N-back task performance in the three language groups

**Fig 2:**
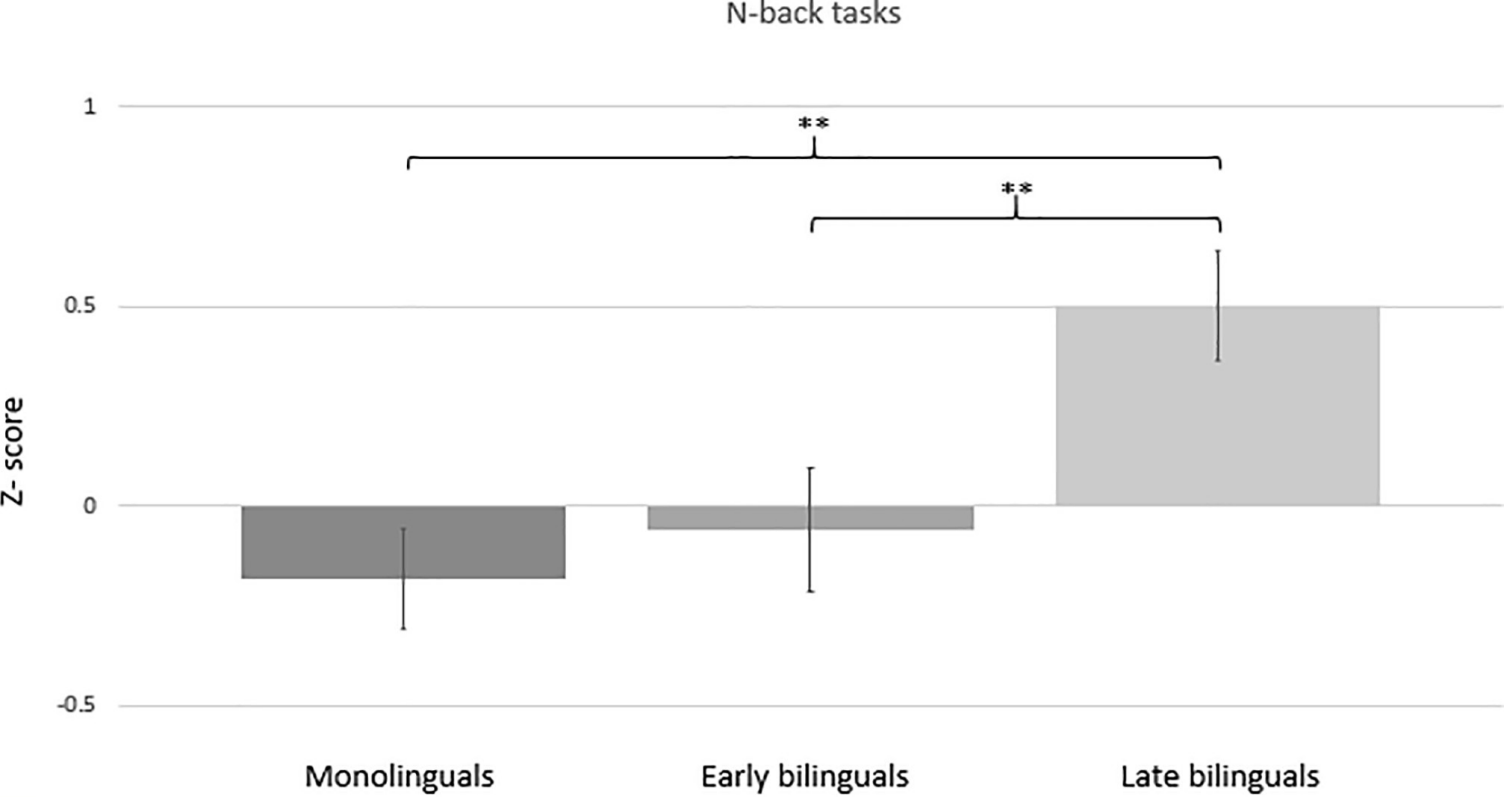
Differences between language groups in the n-back performance. Bars represent standard errors.

ANCOVA with the n-back task composite as the dependent variable, age and education as covariates, and language group (monolingual; early bilingual; late bilingual) as the independent variable, using MatchIt weights as weighted least squares, showed a significant effect of language group [F(2,435) = 7.36, p < .01; partial η^2^ = .033]. This stemmed from an advantage of late bilinguals over monolinguals and early bilinguals. Monolinguals did not differ significantly from early bilinguals (p > .05), but they did differ from late bilinguals (p < .01). The difference between early and late bilinguals was significant as well (p < .01). Moreover, age was a significant covariate (p < .01), with lower age being related to a better performance in the n-back tasks. The group differences are shown in Fig 2. Bayesian ANCOVA yielded a Bayes Factor BF_10_ = 10.98 for language group as compared to a null model with just the covariates, indicating substantial support for the hypothesis that the language groups differ in the n-back task.

The n-back composite analyzed above included both verbal and visuospatial n-back tasks. Given that there was some indication for larger differences between the language groups in visuospatial than in verbal WM, we ran additional analyses separately for these two n-back tasks. For both tasks, ANCOVAs revealed a significant main effect of language group (verbal n-back task: F(2,435) = 4.15, p < .01; visuospatial n-back task: F(2,435) = 5.73, p < .01), and age was a significant covariate, with younger participants achieving higher scores. In the verbal n-back task, late bilinguals were significantly more accurate than monolinguals (p < .05) and early bilinguals (p < .05), while monolinguals did not differ significantly from early bilinguals (p > .05). In the corresponding Bayesian ANCOVA, the Bayes Factor for language group was BF_10_ = 0.28, giving moderate evidence for null hypothesis. Likewise, for the visuospatial n-back task late bilinguals achieved significantly higher accuracy than early bilinguals (p < .05) and monolinguals (p < .01). There was no significant difference between early bilinguals and monolinguals (p > .05). In the Bayesian ANCOVA, the Bayes Factor for language group was BF_10_ = 8.26.

Overall, these results suggest that bilingualism is associated with higher scores in the n-back and visuospatial WM tasks, but the Bayesian analyses supported group differences only for the visuospatial n-back performance. Moreover, in the n-back task, the bilingual advantage was specifically due to higher performance of late bilinguals compared to the other groups. In the next step, we employed a within-group approach to study whether some aspects of bilingual experience were associated with n-back performance, the only WM composite that showed consistent evidence for language group differences. If the BEA hypothesis holds, one would expect that critical aspects of bilingual experience would be associated with performance on an executively loaded measure (here the n-back composite) that at group level has shown a bilingual advantage.

### Associations between bilingual experience and WM performance

In hierarchical regression analyses of predictors of n-back performance, we used a within-subjects design and considered all bilingual participants (both early and late) as one group, and AoA as a continuous variable. Model 1 was our baseline model, including the three general background variables age, education, and childhood socioeconomic status. Model 2 included the additional bilingualism-related factors L2 AoA, L2 proficiency and frequency of language switching, and it was compared to Model 1 which served as the null model in this comparison. Table 5 summarizes both models.

**Table 5:** Hierarchical regression models for general background variables and bilingual language features as predictors of n-back performance.

	Model 1			Model 2		
Variable	B	SE B	β	B	SE B	β
Age	-.02	.01	-.14*	-.03	.01	-.16*
Education	-.08	.09	-.06	-.05	.09	-.04
Childhood SES	.04	.09	.03	.03	.09	.02
L2 AoA				.01	.01	.06
L2 proficiency				-.07	.08	-.06
Language switching				-.16	.17	-.06
Adjusted R^2^	.015			.025		
F for change in R^2^	2.31			1.91		
BF_10_	0.16			0.35		

*p < .01.

Model 1 was not significant (adjusted R^2^ = .015, F(3,261) = 2.314, p = .076). Bayesian analysis supported the null model (BF₁₀ = .16).

Model 2 was not significant either (adjusted R^2^ = .025, F(3,258) = 1.91, p = .13). As in Model 1, there was a negative correlation between age and n-back performance (standardized β = -.17, p = .01). Model 2 was not supported by Bayesian analysis either; it did not have a higher fit than Model 1 (BF₁₀ = .35).

We also conducted the same hierarchical regression analysis for n-back performance in late bilinguals only, prompted by the ANCOVA results where late bilinguals outperformed the other groups. Neither model was significant or supported by Bayesian analysis [Model 1: adjusted R^2^ = .005, F(3,146) = 1.275, p = .285; BF₁₀ = 0.08; Model 2: adjusted R^2^ = 0, F(6,143) = .979, p = .44; BF₁₀ = 0.01].

## Discussion

We investigated the BEA hypothesis in the domain of working memory. WM was measured by three WM composites that were derived from a previous latent factor analysis [43]. We applied two complementary analysis approaches. First, we compared monolingual, early bilingual and late bilingual groups to each other on the three WM measures. Second, we examined whether the WM measure showing a significant bilingual advantage (n-back composite) was associated with some key features of bilingual experience (L2 AoA, L2 proficiency, frequency of language switching) within the bilingual participants. The study was motivated by the fact that most of the existing studies on bilingualism and WM have employed primarily simple span tasks and small samples, and the groups have not necessarily always been fully matched on relevant background variables. We addressed these issues by recruiting a large online sample, matching mono- and bilingual participants on several background variables, using an extensive WM test battery that included more complex tasks, and basing our WM measures on the latent structure of the tasks.

The group comparisons between monolinguals, early bilinguals and late bilinguals showed statistically significant effects for the n-back and visuospatial WM performance, but not for the verbal WM composite. On the other hand, Bayes factors provided evidence for a group difference only in the n-back task, while in the verbal WM composite there was evidence for a *lack* of group difference and in the visuospatial WM composite there was no evidence for either hypothesis. For the n-back, we observed higher scores in the late bilingual group, while early bilinguals were comparable to monolinguals. In visuospatial WM, the difference between early and late bilinguals was not statistically significant, and both the early and late bilingual groups performed better than the monolingual group. It is, however, important to highlight that the observed effect size was quite small (η^2^ = .02) and the Bayes factor did not support the group difference (BF_10_ = 2.46).

Despite this variability, we can conclude that the present results are broadly in line with earlier meta-analyses that have reported small advantages on WM in bilinguals [21, 22], albeit these effects did not survive correction for publication bias in the most recent extensive meta-analysis by Lehtonen and colleagues [16]. Also in line with Lehtonen et al. [16], the effect size appeared larger in nonverbal than in verbal WM. A possible reason for this is that bilinguals, who by definition have had less exposure to each language, show some disadvantages in the verbal domain when compared with monolinguals.

As the present correlative findings cannot establish causality, it could also be that the better WM updating performance (as reflected by the comparatively higher n-back performance) in our late bilinguals reflects higher baseline executive skills. According to this account, well-developed executive abilities would have enabled more efficient L2 learning in our late bilinguals. It has been shown that WM performance is positively correlated with novel word learning (for an overview, see [57]). In their meta-analysis, Linck and colleagues [58] focused specifically on late bilinguals and proficient L2 learners, showing that L2 processing and proficiency measures were positively associated with WM. They hypothesized that people with greater WM capacity are more likely to succeed in learning a second language. In a pre-post longitudinal study with adult L2 learners in classroom settings, Linck and Weiss [59] indeed found that greater WM resources predicted L2 proficiency at posttest 8 weeks later. It could also be that there were some aspects of language learning in the late bilingual group (for example, distinctive language strategies) which promoted WM updating. Furthermore, according to the controlled dose hypothesis recently suggested by Paap ([60], in press), late bilinguals might be experiencing a “boost” in executive performance due to cognitive demands of L2 use which has not yet been automatized (see also [61]). Considering the possible influence of non-linguistic factors, our study would have benefited from employing a non-verbal intelligence measure, as WM updating tasks have been shown to correlate positively with Raven’s matrices scores [45]. Probing the participants’ non-verbal intelligence would have allowed us to control for baseline differences more comprehensively.

We followed up the only clearcut group difference, the late bilinguals’ advantage on the n-back composite, by examining whether some key features of bilingual experience (L2 AoA, L2 proficiency and frequency of language switching) together with demographic factors would predict n-back performance within the bilingual group. Our results were negative for all three variants as the regression models failed to significantly account for variation in n-back performance. Thus, either our measures of bilingual experience were faulty, or the observed group difference in n-back stems from some other, uncontrolled factors than bilingual experience.

Calvo and colleagues [62] have argued that bilingualism-related WM benefits may be specific to some components of WM only, being easily overshadowed by the great variability in WM tasks and the common use of verbal WM measures which can be disadvantageous to bilinguals. However, this idea does not quite fit to our finding that the only clearcut group difference concerned an updating composite including both verbal and visuospatial task variants, and that the variability on this composite was not explained by bilingualism-related factors. Yet another possible variant of the BEA hypothesis is that the type of bilingual experience may be crucial for the emergence of BEA. According to the Adaptive Control hypothesis put forth by Green and Abutalebi [63], the executive load (and thus the executive practice provided by bilingualism) depends on the type of communicative context that a bilingual person usually lives in. More specifically, they argue that a dual-language context where each language is used with different communication partners would tax executive functions most heavily. In contrast, dense code-switching and also single-language context would involve less executive load. Thus, it is possible that many of our bilinguals had not lived in the cognitively most demanding dual-language communicative context. It is also possible that the responses to our language switching question reflect a mixture of switches in dense code-switching and dual-language contexts, thus weakening our chances for observing a correlation between WM and switching frequency.

In summary, despite some group differences favoring bilinguals, our study failed to find consistent support for the BEA hypothesis in the domain of working memory. A major difficulty in this venue of research is that the original hypothesis does not delineate the exact conditions of bilingual experience that are required for BEA to emerge. So far, most studies on bilingualism have focused on comparing bilinguals to monolinguals, with less attention given to individual differences within the bilingual groups. If bilingual experience enhances EF, it is important to specify what aspects of this experience are beneficial. Future studies will show whether a more stringent version of the BEA hypothesis, such as the one based on the Adaptive Control model by Green and Abutalebi [63], would provide consistent, replicable effects. Meanwhile, the null hypothesis should prevail.

## Supporting information

S1 Table Data used for the analysis. (RAR)
